# Genetic Diversity and Population Structure of the Pelagic Thresher Shark (*Alopias pelagicus*) in the Pacific Ocean: Evidence for Two Evolutionarily Significant Units

**DOI:** 10.1371/journal.pone.0110193

**Published:** 2014-10-22

**Authors:** Diego Cardeñosa, John Hyde, Susana Caballero

**Affiliations:** 1 Laboratorio de Ecología Molecular de Vertebrados Acuáticos-LEMVA, Departamento de Ciencias Biológicas, Universidad de Los Andes, Bogotá, Colombia; 2 Southwest Fisheries Science Center, National Marine Fisheries Service, La Jolla, California, United States of America; Simon Fraser University, Canada

## Abstract

There has been an increasing concern about shark overexploitation in the last decade, especially for open ocean shark species, where there is a paucity of data about their life histories and population dynamics. Little is known regarding the population structure of the pelagic thresher shark, *Alopias pelagicus*. Though an earlier study using mtDNA control region data, showed evidence for differences between eastern and western Pacific populations, the study was hampered by low sample size and sparse geographic coverage, particularly a lack of samples from the central Pacific. Here, we present the population structure of *Alopias pelagicus* analyzing 351 samples from six different locations across the Pacific Ocean. Using data from mitochondrial DNA COI sequences and seven microsatellite loci we found evidence of strong population differentiation between western and eastern Pacific populations and evidence for reciprocally monophyly for organelle haplotypes and significant divergence of allele frequencies at nuclear loci, suggesting the existence of two Evolutionarily Significant Units (ESU) in the Pacific Ocean. Interestingly, the population in Hawaii appears to be composed of both ESUs in what seems to be clear sympatry with reproductive isolation. These results may indicate the existence of a new cryptic species in the Pacific Ocean. The presence of these distinct ESUs highlights the need for revised management plans for this highly exploited shark throughout its range.

## Introduction

Sharks of the open ocean are wide-ranging, highly migratory species that routinely cross national borders. Such characteristics may lead to the misconception that these marine resources are unlimited and resistant to localized depletion. When population subdivision exists, these misconceptions lead to an unsustainable use of the resource, ending in the depletion of the population under pressure. If severe enough, these population depletions may result in reduced genetic diversity and a concomitant reduction in the ability of a population to adapt to environmental or disease stressors [1], [2]. To identify management units a variety of tools are available for sharks (e.g. conventional and electronic tagging, life history studies, population genetic analyses, fishery catch data). Over the last 20 years genetic data have become increasingly important for delineating management units and understanding population connectivity in the marine realm.

Highly migratory and broadly distributed open ocean shark species are expected to show little to no population heterogeneity. As expected, previous population genetic studies on epipelagic sharks, such as the shortfin mako shark *Isurus oxyrinchus* [3], the basking shark *Cetorhinus maximus* [4], the whale shark *Rhincodon typus* [5], [6], and the blue shark *Prionace glauca* [7] show low to no genetic structuring among ocean basins.

The pelagic thresher shark (*Alopias pelagicus*) is a large (up to 330 cm TL) aplacental viviparous epipelagic shark with a distribution restricted to the Indian and Pacific Oceans. Fecundity is very low with litters of only 1–2 pups [8] with an unknown gestation period, but assumed to be around a year or less [9]. The pelagic thresher shark is one of the most abundant open ocean sharks in the Eastern Tropical Pacific (ETP) and Western Pacific Ocean (WP), and one of the most exploited shark species in commercial, artisanal and illegal fisheries of these regions [10]–[12]. Despite a high level of exploitation little is known regarding their life history, population dynamics, and overall abundance.

Between 2009 and 2011, tissue samples, collected in Colombian fishing ports and during seizures of illegal shark finning vessels, were sent to the Universidad de Los Andes for molecular identification. We found that more than 95% of those samples were *A. pelagicus*, suggesting that perhaps exploitation rates in this region are higher than previously thought and that there is a need to gather more information on this species for its effective management [12]. Furthermore, Tsai et al. [11] suggested, using a stochastic stage-based model, that the northwest Pacific stock of *A. pelagicus* is overexploited. The combined levels of exploitation, low fecundity and lack of population information led this species to be classified as Vulnerable on the IUCN Red List.

Despite their common occurrence and importance to fisheries, little is known about the genetic diversity of thresher sharks. Early work by Eitner [13], used allozyme data to examine phylogenetic relationships among the three species and suggested the existence of an unrecognized taxon in the Eastern Pacific. Trejo [14] studied the global population structure and genetic diversity of all three thresher shark species by analyses of DNA sequence data from the mitochondrial control region, finding different phylogeographic patterns for each species. Genetic heterogeneity was present among sampled populations of *A. vulpinus* but with no clear biogeographic signal. Slight population differentiation existed between samples of *A. superciliosus* from locations in the Atlantic and the Indo-Pacific, but not among samples from within the Indo-Pacific. In contrast to the other two species, *A. pelagicus* populations exhibited a very marked biogeographic pattern with strong population structure between Eastern and Western Pacific locations indicating limited gene flow across the Pacific Ocean. An interesting finding was the presence of two well-differentiated clades corresponding roughly to sampling region. One haplotype clade representing samples predominantly from the Western Pacific and the other grouping haplotypes found exclusively in the Eastern Pacific.

Though a strong biogeographic signal was detected for *A. pelagicus*, Trejo's study was limited by the number of samples both overall and within sampled areas, by a lack of geographically intermediate samples from the Central Pacific, and by the use of a single mitochondrial marker. Despite their limitations, the results of both Trejo [14] and Eitner [13] identify interesting patterns of genetic heterogeneity that deserve further evaluation. Here, we build upon previous examination of the molecular ecology of *A. pelagicus* through increased sample number, inclusion of additional sampling areas spanning the range of *A. pelagicus* in the Pacific Ocean, and analyses of both mitochondrial and nuclear molecular markers. The generated data will allow us to evaluate several key questions: 1.) Are the patterns observed by Trejo similar when bi-parental nuclear data are included? 2.) Given the strong vicariance between Eastern and Western Pacific locations observed by Trejo [14], are intermediate haplotypes present in the Central Pacific? 3.) Are the distinct clades identified by Trejo [14] more likely the result of population subdivision with restricted geneflow or do they support the existence of an unrecognized taxon as suggested by Eitner [13]?

## Methodology

### Tissue collection and DNA extraction

**Figure 1:**
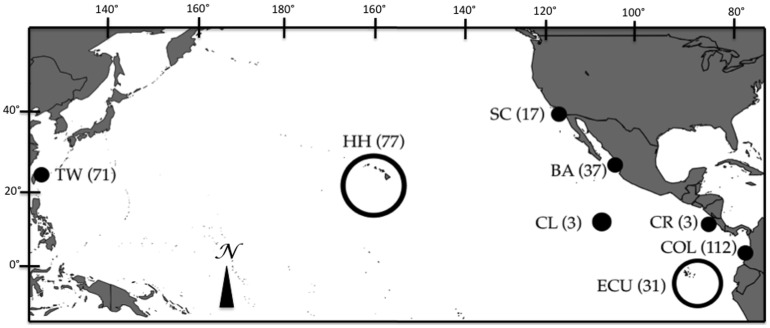
Map indicating sampling locations in the Pacific Ocean. TW = Taiwan, HH = Hawaii, BA = Baja, SC = Southern California, CL = Clipperton Island, CR = Costa Rica, COL = Colombia, ECU = Ecuador. Sample numbers for each location are shown in parentheses.

A total of 351 samples were collected between 1997 and 2011 (Fig. 1). The majority of these samples were collected by fishery observers from dead animals captured in regional fisheries using a variety of different capture techniques (i.e. longline, purse seine, gillnet). Additional samples were collected during NOAA research cruises and from sampling landings made by artisanal fishers. Collaborating institutions that aided in the collection of samples used in this study include; the National Marine Fisheries Service (USA), Incoder Subdirección de Pesca (Colombia), the Inter-American Tropical Tuna Commission, the Ministerio de Agricultura, Ganaderia, Acuacultura, y Pesca (Ecuador), the Fisheries Research Institute (Taiwan). No Ethical Approval was required for this project since the samples were collected from dead animals already captured in commercial and recreational fishing activities.

Samples were identified by experts in the field and for the samples for which identification was uncertain, we implemented the molecular identification protocol described by Caballero *et al.* [12]. DNA was extracted by heating small pieces of tissue in 200 µl of 10% Chelex solution (BioRad) at 60°C for 20 minutes, then 103°C for 25 minutes followed by a brief centrifugation and storage at 4°C [15].

### Cytochrome Oxidase I amplification and analyses

A portion of the mitochondrial COI gene (655 bp) was amplified using the universal primers FishCoxI F (5′ TCWACCAACCACAAAGAYATYGGCAC) and FishCoxI R (TARACTTCWGGGTGRCCRAAGAATCA) modified from Ward *et al.* [16]. The PCR profile was as follows: 94°C for 2 min followed by 35 cycles of 94°C for 30 s, 55°C for 45 s and 72°C for 40 s, with a final extension of 72°C for 10 min. A no-template negative control was included with each PCR batch to monitor for reagent contamination. PCR products were visualized on 2% agarose gels stained with ethidium bromide and single-band PCR products were enzymatically cleaned using ExoSapIT (Affymetrix). Cleaned products were cycle sequenced using BigDye Terminator v3.1 chemistry and run on an ABI3730 Genetic Analyzer.

All forward and reverse sequences were checked and edited manually using Geneious Pro v. 3.6.1 (http://www.geneious.com/) and aligned using Seaview 4.4.1 software [17]. Haplotypes were defined using MacClade [18]. A statistical parsimony network was constructed using the software TCS v. 1.21 [19], providing a 95% plausible set for all haplotype linkages. The optimal model of DNA substitution was chosen using JModelTest v.2.3.1 [20]. To understand phylogenetic patterns among haplotypes the best fit model (GTR+I) was used for phylogenetic reconstructions performed in Beast v.1.7.5 [21].

Haplotype and nucleotide diversity calculations as well as pairwise comparisons of both F_ST_ and Φ_ST_ were performed using Arlequin v.3.5.1 [22] using the pairwise nucleotide difference model to calculate genetic distance. As Trejo [14] showed evidence for two distinct mtDNA clades and Eitner [13] suggested the existence of a possible unrecognized taxon, we chose to partition the samples in two distinct manners. In the first partition, samples were grouped solely by geographic region (i.e. Taiwan, Hawaii, southern California, Baja, Central America, Colombia, Ecuador). In the second partition, samples were first grouped by the mtDNA clade their COI haplotype belonged to (i.e. Clade A, Clade B) and subsequently grouped by geographic region. Analysis of molecular variance (AMOVA), using 10,000 random permutations, was performed to identify optimal groupings and to test biogeographic and phylogenetic hypotheses.

### Microsatellite loci amplification and analyses

Nine microsatellite loci (Iox-01, Iox-30, Iox-12 [3]) and (AV-H8, AV-H110, AV-H138, AV-I11, Iox-M36 and Iox-M115). Primer sequence and repeat information for primers AV-H8, AV-H10, AV-H138, AV-111, Iox-M36 and Iox-M115 are available on GenBank (Table S1). Microsatellite loci were amplified separately in 30 µl PCR reactions. Two thermocycling profiles were implemented. The first profile consisted of an initial denaturation at 94°C for 2 min, followed by 35 cycles of 94°C for 30 s, 60 s at specified annealing temperature (Table S1), and 72°C for 90 s, with a final extension of 72°C for 30 min. The second profile consisted of the same temperatures with a final extension of 60 min (Table S1).

PCR products were run on either an ABI 3500 automated sequencer at Universidad de Los Andes (Colombia) or an ABI 3730 Genetic Analyzer at the NOAA SWFSC (La Jolla, CA) using the internal ROX 500 size standard. Electropherograms were visualized and allele size calling was done using ABI PRISM GeneMapper Software v4.1 (Life Technologies). A reference set of samples were used to cross-calibrate scores between analysis platforms and labs. All authors scored all samples for all loci individually in order to minimize scoring errors across readers.

Patterns of genetic structure were evaluated using Structure v2.3.4 [23], [24], which assigns individuals to groups using a Bayesian model-based method that minimizes linkage disequilibrium and deviations from Hardy-Weinberg expectations. The admixture model with correlated allele frequencies was selected and models were run that both did and did not employ the sampling location prior [25]. The sampling location prior was used in two distinct ways. To better elucidate localized genetic structure individuals were coded by region of capture, using it as a prior. Additionally, to test for possible reproductive isolation between the two mtDNA clades identified by Trejo [14], individuals were coded by which mtDNA clade their COI haplotype belonged to using both geographic location and mtDNA clade as priors in this analysis. To infer the most likely number of groups (K) we compared the log probability LnP(D) of different values for K using an *ad hoc* statistic (Δ*K*, [26]) that calculates the second order rate of change of LnP(D). Runs were performed with a 10,000 step burn-in followed by 100,000 MCMC steps to test *K* = 1–10 with 20 repetitions each.

To test for population differentiation and genetic diversity, samples with three or more loci with missing data were excluded. Differentiation and genetic diversity among population units were assessed utilizing several methods. Pairwise F_ST_ and R_ST_ values were calculated using Arlequin v.3.5.1 [22], population differentiation was tested by exact test as implemented in Genepop [27], and a genetic differentiation index D [28] was calculated using the R-package DEMEtics [29]. Departures from expectations of both linkage disequilibrium and Hardy-Weinberg equilibrium (HWE) and were tested for using Arlequin v.3.5.1 [22].

## Results

### Mitochondrial DNA COI analyses

**Figure 2:**
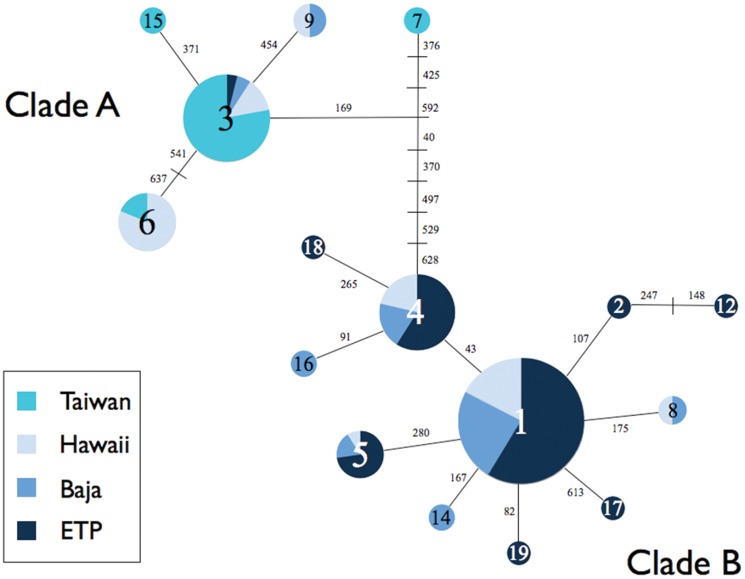
Haplotype network obtained from the TCS analysis. The size of the circle represents the frequency of each haplotype. Numbers represent substitutions between haplotypes and small lines represent hypothetical haplotypes not observed in this study.

**Figure 3:**
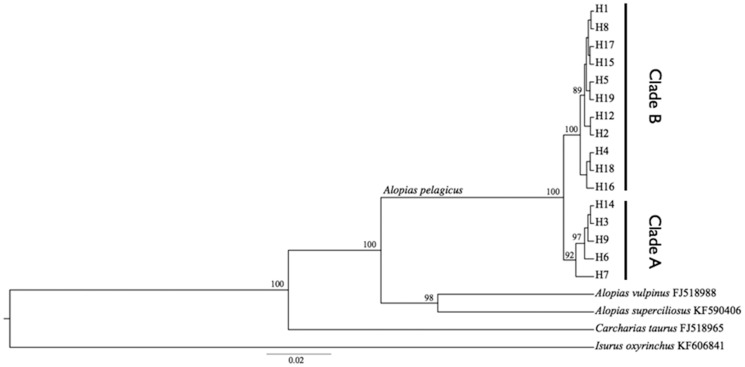
Coalescent tree showing reciprocal monophyly between haplotypes from the A and B clades. Posterior values are shown as branch labels.

A total of 323 sequences of the 655 bp COI fragment were successfully obtained from samples analyzed. Analysis of these data identified 24 variable sites that defined 19 unique haplotypes, although H11 was removed due to excessive missing data (Table 1), and H10 and H13 were removed from TCS and Beast analyses for the same reason. All haplotype sequences were submitted to GenBank under accession numbers KM218907–KM218923. The haplotype network derived from the TCS analysis and a phylogenetic tree showed two well differentiated and well supported clusters, hereafter referred to as clade A and clade B (Figures 2 and 3). Nine haplotypes were unique to individual regions (H2, H12, H17, H18, and H19 in the ETP region, H14 and H16 in Baja, and H7 and H15 in Taiwan) with the rest being observed across multiple regions. Haplotype H1 showed the highest frequency in the eastern Pacific, and the analysis suggested it to be the ancestral haplotype in this region. Haplotype H3 was the most common in the western Pacific and only seven samples from the eastern Pacific shared that haplotype. Haplotype H9 was shared between Hawaii and Baja. Of the sequences obtained from GenBank, the Indo-Pacific samples grouped within clade A and the single sample from Mexico grouped within clade B. Sampling locations at the edge of the sampling distribution (Taiwan and ETP) were composed entirely of haplotypes from a single clade, clade A haplotypes in Taiwan and clade B haplotypes in the ETP. Samples from Baja and Hawaii contained a mixture of haplotypes from both clades (Figure 2).

**Table 1:** Twenty-four variable sites over 655 bp of the mitochondrial COI gene determining 19 Pacific *Alopias pelagicus* haplotypes.

	Variable Sites																							
Haplotypes	40	43	82	91	107	145	167	169	175	247	265	280	370	371	376	425	454	497	529	541	592	613	628	637
H1	G	G	A	T	A	A	G	A	T	T	T	T	G	G	C	T	T	C	C	A	C	T	G	T
H2	.	.	.	.	G	.	.	.	.	.	.	.	.	.	.	.	.	.	.	.	.	.	.	.
H3	A	A	.	.	.	.	.	G	.	.	.	.	A	.	.	.	.	T	T	.	.	.	A	.
H4	.	A	.	.	.	.	.	.	.	.	.	.	.	.	.	.	.	.	.	.	.	.	.	.
H5	.	.	.	.	.	.	.	.	.	.	.	C	.	.	.	.	.	.	.	.	.	.	.	.
H6	A	A	.	.	.	.	.	G	.	.	.	.	A	.	.	.	.	T	T	G	.	.	A	C
H7	A	A	.	.	.	.	.	.	.	.	.	.	A	.	T	C	.	T	T	.	T	.	A	.
H8	.	.	.	.	.	.	.	.	C	.	.	.	.	.	.	.	.	.	.	.	.	.	.	.
H9	A	A	.	.	.	.	.	G	.	.	.	.	A	.	.	.	C	T	T	.	.	.	A	.
H10	?	?	?	?	?	.	.	.	.	.	.	.	.	.	.	.	.	.	.	.	.	.	.	.
H12	.	.	.	.	G	G	.	.	.	C	.	.	.	.	.	.	.	.	.	.	.	.	.	.
H13	.	.	.	.	.	.	.	.	.	.	.	.	.	.	.	.	.	.	.	.	.	C	?	?
H14	A	A	.	.	.	.	.	G	.	.	.	.	A	A	.	.	.	T	T	.	.	.	A	.
H15	.	.	.	.	.	.	A	.	.	.	.	.	.	.	.	.	.	.	.	.	.	.	.	.
H16	.	A	.	C	.	.	.	.	.	.	.	.	.	.	.	.	.	.	.	.	.	.	.	.
H17	.	.	.	.	.	.	.	.	.	.	.	.	.	.	.	.	.	.	.	.	.	C	.	.
H18	.	A	.	.	.	.	.	.	.	.	C	.	.	.	.	.	.	.	.	.	.	.	.	.
H19	.	.	G	.	.	.	.	.	.	.	.	.	.	.	.	.	.	.	.	.	.	.	.	.

(?) denotes missing data.

Comparisons of F_ST_ and Φ_ST_ between geographically sampled populations produced very high values of Φ_ST_ for most pairwise comparisons (Φ_ST_ 0.0624–0.8964) with most populations being significantly different from each other (Table 2), though comparison between Baja and ETP populations was not significant after Bonferroni correction. When comparisons were performed separating locations by mtDNA clade, comparison of clade A populations produced a broad range of values of Φ_ST_ (Φ_ST_ 0.0768–0.4971) with significant differentiation for all comparisons involving Baja (Table 3). Baja had a very low clade A sample size so the power to differentiate this population is low and values of F_ST_ and Φ_ST_ are likely inaccurate. For the comparisons involving only Clade B populations, we found low values of Φ_ST_ (Φ_ST_ −0.0066–0.0131) and non-significant differences in pairwise comparisons for both F_ST_ and Φ_ST_ (Table 4).

**Table 2:** Pairwise Fst (below diagonal) and Φst (above diagonal) values for the COI gene from the Pacific Ocean populations of *Alopias pelagicus*.

FST ΦST	ETP (n = 121)	Baja (n = 56)	Hawaii (n = 75)	Taiwan (n = 64)
ETP	π = 0.104% h = 0.586±0.036	0.0624 (*0.0014±0.000*)	0.4766 **(** ***0.000±0.000*** **)**	0.8964 **(** ***0.000±0.000*** **)**
Baja	−0.0026 (*0.432±0.005*)	π = 0.299% h = 0.631±0.061	0.2608 **(** ***0.000±0.000*** **)**	0.7882 **(** ***0.000±0.000*** **)**
Hawaii	0.1458 **(** ***0.000±0.000*** **)**	0.1131 **(** ***0.000±0.000*** **)**	π = 0.688% h = 0.755±0.024	0.36255 **(** ***0.000±0.000*** **)**
Taiwan	0.5283 **(** ***0.000±0.000*** **)**	0.5092 **(** ***0.000±0.000*** **)**	0.3728 **(** ***0.000±0.000*** **)**	π = 0.130% h = 0.306±0.071

Probability values based on 10,000 permutations are shown in italic. Significant scores after Bonferroni correction are in bold. Haplotype (h) and nucleotide (π) % ± standard deviation (SD) diversity values are shown in the diagonal of each population unit. Numbers of samples per location are shown in parentheses.

**Table 3:** Pairwise Fst (below diagonal) and Φst (above diagonal) values for the COI gene when only Clade A haplotypes of *Alopias pelagicus* are analyzed.

FST ΦST	Baja (n = 6)	Hawaii (n = 38)	Taiwan (n = 71)
Baja	π = 0.081% h = 0.533±0.172	0.4971 **(** ***0.000±0.000*** **)**	0.4355 **(** ***0.000±0.000*** **)**
Hawaii	0.3916 **(** ***0.003±0.001*** **)**	π = 0.151% h = 0.472±0.072	0.0768 (*0.111±0.003*)
Taiwan	0.4728 **(** ***0.000±0.000*** **)**	0.1239 (*0.079±0.003*)	π = 0.130% h = 0.306±0.071

Probability values based on 10,000 permutations are shown in italic. Significant different values (p<0.05) in bold. Haplotype (h) and nucleotide (π) % ± standard deviation (SD) diversity values are shown in the diagonal of each population unit. Numbers of samples per location are shown in parentheses.

**Table 4:** Pairwise Fst (below diagonal) and Φst (above diagonal) values for the COI gene when only Clade B haplotypes of *Alopias pelagicus* are analyzed.

FST ΦST	ETP (n = 121)	Baja (n = 47)	Hawaii (n = 37)
ETP	π = 0.104% h = 0.586±0.036	−0.0092 (*0.788±0.004*)	−0.0131 *(0.666±0.005)*
Baja	−0.0085 (*0.679±0.005*)	π = 0.095% h = 0.536±0.066	−0.0066 (*0.554±0.005*)
Hawaii	−0.0128 (*0.619±0.005*)	−0.0104 (*0.691±0.004*)	π = 0.088% h = 0.536±0.055

Probability values based on 10,000 permutations are shown in italic. Significant different values (p<0.05) in bold. Haplotype (h) and nucleotide (π) % ± standard deviation (SD) diversity values are shown in the diagonal of each population unit. Numbers of samples per location are shown in parentheses.

Haplotype diversity was similar in each sampled area with the highest nucleotide diversity found in Hawaii. Taiwan showed the lowest haplotype diversity compared to all other regions (Table 3).

### Microsatellite analyses

Microsatellite fragments were analyzed for nine microsatellite loci for 331 individuals. Initial scoring identified two loci, Iox-01 and Iox-30 that were monomorphic in this species and were subsequently eliminated from downstream analyses. The number of alleles ranged between two (AV-I11) and 39 (AV-H8) across all sampled populations.

Genetic diversity values including expected (H_E_) and observed heterozygosity (H_O_), were obtained for seven loci in all populations units, along with deviations from H-W equilibrium (Table 5). H_E_ and H_O_ varied among population units at different loci. When samples were partitioned strictly by location, HWE analyses showed the Hawaii and Baja regions were significantly out of HWE at most loci while the Taiwan and ETP regions were mostly in HWE. The alternate sample partitioning that separated out clade A and B individuals by location showed groups to be in HWE for all loci except locus AVH8 in the sample set from Colombia and locus Iox-12 in samples from southern California (Table 5).

**Table 5:** Genetic diversity for seven microsatellite loci in all sampling locations analyzed.

loci	TW	HHWP	HHEP	BA	SC	CA	COL	ECU
	N = 71	N = 32	N = 36	N = 10	N = 18	N = 5	N = 92	N = 31
	n = 18	n = 15	n = 23	n = 14	**n = 14**	n = 8	n = 36	n = 25
Iox-12	Ho = 0.929	Ho = 0.813	Ho = 0.778	Ho = 1.00	**Ho = 0.529**	Ho = 0.800	Ho = 0.900	Ho = 0.900
	He = 0.918	He = 0.897	He = 0.941	He = 0.963	**He = 0.941**	He = 0.956	He = 0.953	He = 0.953
	p = 0.0283	p = 0.3899	p = 0.0098	p = 1.000	**p = 0.0000**	P = 0.262	p = .0075	p = 0.0574
	n = 3	n = 3	n = 3	n = 2	n = 2	n = 2	n = 2	n = 3
Iox-M36	Ho = 0.471	Ho = 0.219	Ho = 0.265	Ho = 0.111	Ho = 0.056	Ho = 0.000	Ho = 0.315	Ho = 0.138
	He = 0.405	He = 0.249	He = 0.415	He = 0.111	He = 0.056	He = 0.533	He = 0.360	He = 0.194
	p = 0.2479	p = 0.4805	p = 0.0419	p = 1.000	p = 1.000	p = 0.0474	p = 0.2489	p = 0.1061
	n = 3	n = 2	n = 2	n = 2	n = 2	n = 2	n = 2	n = 3
AV-I11	Ho = 0.296	Ho = 0.188	Ho = 0.472	Ho = 0.500	Ho = 0.222	Ho = 0.400	Ho = 0.609	Ho = 0.581
	He = 0.306	He = 0.222	He = 0.488	He = 0.395	He = 0.514	He = 0.533	He = 0.494	He = 0.539
	p = 0.4527	p = 0.3912	p = 1.000	p = 1.000	p = 0.0215	P = 1.000	p = 0.0328	p = 0.0161
	n = 31	n = 25	n = 29	n = 12	n = 19	n = 6	**n = 39**	n = 21
AV-H8	Ho = 0.939	Ho = 0.931	Ho = 0.879	Ho = 0.778	Ho = 0.875	Ho = 0.800	**Ho = 0.965**	Ho = 0.900
	He = 0.953	He = 0.960	He = 0.971	He = 0.922	He = 0.958	He = 0.844	**He = 0.967**	He = 0.953
	p = 0.2991	p = 0.4292	p = 0.0957	p = 0.0562	p = 0.0152	p = 0.7962	**p = 0.0009**	p = 0.0025
	n = 16	n = 10	n = 19	n = 9	n = 11	n = 9	n = 32	n = 24
AV-H138	Ho = 0.958	Ho = 0.750	Ho = 0.861	Ho = 0.900	Ho = 0.944	Ho = 0.800	Ho = 0.868	Ho = 0.935
	He = 0.872	He = 0.826	He = 0.889	He = 0.826	He = 0.887	He = 0.978	He = 0.902	He = 0.937
	p = 0.3662	p = 0.0952	p = 0.4408	p = 0.9976	p = 0.4609	p = 0.1204	p = 0.1349	p = 0.0481
	n = 16	n = 15	n = 18	n = 10	n = 16	n = 5	n = 21	n = 18
AV-H110	Ho = 0.9	Ho = 0.903	Ho = 0.944	Ho = 0.700	Ho = 0.938	Ho = 0.800	Ho = 0.921	Ho = 0.900
	He = 0.913	He = 0.888	He = 0.908	He = 0.916	He = 0.952	He = 0.822	He = 0.906	He = 0.905
	p = 0.5505	p = 0.4710	p = 0.9680	p = 0.1221	p = 0.7795	p = 0.8999	p = 0.1407	p = 0.1942
	n = 9	n = 8	n = 8	n = 5	n = 5	n = 4	n = 8	n = 7
Iox-M115	Ho = 0.818	Ho = 0.667	Ho = 0.548	Ho = 0.500	Ho = 0.333	Ho = 1.00	Ho = 0.698	Ho = 0.643
	He = 0.808	He = 0.812	He = 0.644	He = 0.683	He = 0.601	He = 0.733	He = 0.718	He = 0.641
	p = 0.3055	p = 0.2376	p = 0.1459	p = 0.3804	p = 0.0388	p = 0.3956	p = 0.1785	p = 0.7269

N = sample size for each population; n = total number of alleles. Ho = observed heterozygosity. He = expected heterozygosity. Significant scores after Bonferroni correction for loci out of equilibrium are shown in bold.

**Figure 4:**
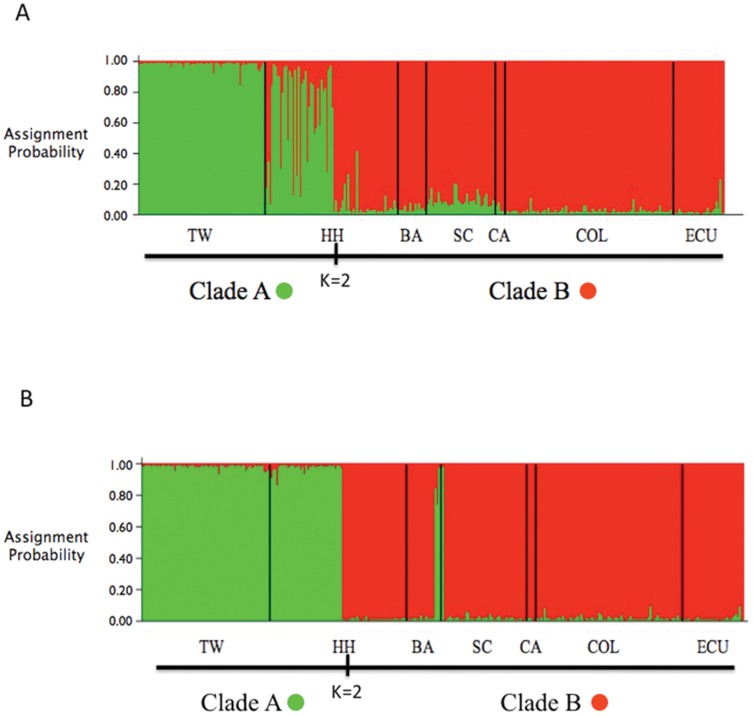
Structure bar plot showing the assignment probabilities (K = 2) of each genotyped individual of *A. pelagicus* from the different sampling locations in the Pacific Ocean. A) sampling region used as location prior. B) COI haplotype clade (A or B) used as the location prior.

Evaluation of the K values produced by Structure using the Δ*K* method [26] identified K = 2 as the most likely number of groups present in the data both with and without consideration of the location prior used for either geographic location or mtDNA clade (Figure 4a, 4b and Figure 1S). In both Baja and Hawaii where the samples were composed of a mixture of individuals from both clades, use of the clade ID as the location prior resulted in very high assignment of individuals to their respective group (Figure 4a and 4b).

When samples were grouped strictly by location, F_ST_ (0.000–0.075) and R_ST_ (−0.032–0.135) comparisons were significant for most comparisons with Taiwan and between Hawaii and Colombia and Ecuador (Table 6). After separating samples by region and haplotype clade, all F_ST_ (−0.003–0.109) and R_ST_ (−0.032–0.338) comparisons remained large and significant between clade A and B groups while comparisons within clade B group were low and not significant (Table 7). Comparisons within the clade A (e.g. Taiwan and Hawaii Clade A) were significant for F_ST_ and non-significant for R_ST_ values (Table 7). Non-significant values of R_ST_ between Taiwan, Baja, southern California and Central America were found, though this may be an artifact of low sample size for some of these comparisons (Table 6, 7).

**Table 6:** Population differentiation based on Fst and Rst values between pairwise populations with seven microsatellite loci.

	TW	HH	BA	SC	CA	COL	ECU
TW (n = 71)	-----	**0.037**	0.124	0.038	0.231	**0.115**	**0.135**
HH (n = 68)	**0.020**	-----	0.101	0.007	0.199	**0.071**	**0.081**
BA (n = 13)	**0.066**	0.018	-----	0.042	−0.011	−0.019	−0.009
SC (n = 18)	**0.043**	0.013	0.024	-----	0.130	0.036	0.027
CA (n = 5)	**0.075**	0.039	0.045	0.059	-----	0.003	−0.032
COL(n = 91)	**0.047**	**0.008**	0.009	0.009	0.021	-----	−0.014
ECU(n = 31)	**0.041**	0.009	0.017	−0.000	0.037	0.003	-----

Significant scores after Bonferroni correction are in bold and probability values are based on 10,000 permutations. Below diagonal Fst values and above diagonal Rst values. Numbers of samples per location are shown in parentheses.

**Table 7:** Population differentiation based on Fst and Rst values between pairwise populations with seven microsatellite loci with populations split by mtDNA clade.

	TW (A)	HH (A)	HH (B)	BA (B)	SC (B)	CA (B)	COL (B)	ECU (B)
TW (A) (n = 71)	-----	0.008	**0.087**	0.171	0.038	0.231	**0.115**	**0.135**
HH (A) (n = 32)	**0.012**	-----	**0.118**	**0.277**	0.092	**0.338**	**0.150**	**0.156**
HH (B) (n = 36)	**0.056**	**0.063**	-----	0.098	0.001	0.093	0.018	0.020
BA (B) (n = 10)	**0.094**	**0.100**	0.013	-----	0.103	0.014	0.021	0.023
SC (B) (n = 18)	**0.043**	**0.053**	0.012	0.027	-----	0.130	0.036	0.027
CA (B) (n = 5)	**0.075**	**0.109**	0.009	0.070	0.059	-----	0.003	−0.032
COL (B) (n = 91)	**0.047**	**0.055**	−0.003	0.020	0.009	0.021	-----	−0.014
ECU (B) (n = 31)	**0.041**	**0.046**	0.007	0.025	0.000	0.037	0.003	-----

Significant scores after Bonferroni correction are in bold and probability values are based on 10,000 permutations. Below diagonal Fst values and above diagonal Rst values. Mitochondrial DNA clade and numbers of samples per location are shown in parentheses.

As both F_ST_ and R_ST_ are inappropriate measures at deeper levels of divergence, both Jost's D and exact test analyses were performed (Table 8). Jost's D showed a high degree of differentiation between clade A and B groups (0.151–0.357) as well as within clade A locations (0.089). The exact test results identified additional differentiation within clade B locations (Table 8).

**Table 8:** Population differentiation based on Jost's D and Exact test values between pairwise populations with seven microsatellite loci.

	TW (A)	HH (A)	HH (B)	BA (B)	SC (B)	CA (B)	COL (B)	ECU (B)
TW (A) (n = 71)	-----	**+**	**++**	**++**	**++**	**++**	**++**	**++**
HH (A) (n = 32)	**0.089 0.079±0.148**	-----	**++**	**++**	**++**	**++**	**++**	**++**
HH (B) (n = 36)	**0.245 0.202±0.274**	**0.158 0.118±0.216**	-----	−	−	−	−	−
BA (B) (n = 10)	**0.303 0.209±0.367**	**0.189 0.082±0.277**	0.065 −0.049±0.149	-----	−	**+**	**+**	−
SC (B) (n = 18)	**0.213 0.187±0.293**	**0.190 0.105±0.248**	0.036 0.015±0.154	0.144 0.032±0.281	-----	**+**	−	−
CA (B) (n = 5)	**0.338 0.159±0.496**	**0.357 0.239±0.576**	0.085 −0.087±0.251	0.334 0.109±0.544	0.237 0.095±0.515	-----	−	−
COL(B) (n = 92)	**0.2110.226±0.269**	**0.151 0.122±0.195**	0.002 0.022±0.088	0.136 0.059±0.266	0.031 −0.021±0.097	0.150 0.052±0.41	-----	**+**
ECU(B) (n = 31)	**0.2410.205±0.275**	**0.167 0.149±0.251**	0.027 −0.016±0.082	0.141 0.038±0.239	0.010 −0.005±0.1448	0.168 −0.040±0.345	0.004 0.027±0.099	-----

Significant scores after Bonferroni correction are in bold. Below diagonal Jost's D values with 95% confidence intervals and above diagonal exact test significance. Degrees of significance: p<0.0018 (+). p<0.000001 (++). Mitochondrial DNA clade and numbers of samples per location are shown in parentheses.

### Comparisons between sample partitions

Structure likelihood values were compared over multiple K's both with no location prior as well as considering the location prior for both data partitions (geographic location and mtDNA clade). In all analyses K = 2 had the highest likelihood (Table S2). Among the three run variants for K = 2, utilization of the mtDNA clade as location prior had the highest likelihood (−8756.49+−7.88) followed by geographic location as location prior (−8774.73+−7.26) (Table S2).

AMOVA comparisons of microsatellite data were performed to compare hypothesized groups (Table 9). Grouping samples by geographic region (Western Pacific, Central Pacific, Eastern Pacific) explained 1.8% of the variance (Φ_CT_ = 0.018 p = NS), but significant within group variance remained (Φ_SC_ = 0.018 p<0.05). Samples grouped by mtDNA clade (i.e. clade A and B) explained 4.9% of the variance (Φ_CT_ = 0.049 p<0.05), while within group variance was fairly low (Φ_SC_ = 0.008 p<0.05) but still significant.

**Table 9:** AMOVA results for both mtDNA and microsatellite data for alternative grouping of samples.

Groups	mtDNA			Microsatellites		
	Φct	Φsc	Φst	Φct	Φsc	Φst
Regional Grouping	0.643	**0.009**	**0.646**	0.018	**0.023**	**0.041**
mtDNA Clade Grouping	**0.879**	**0.190**	**0.902**	**0.049**	**0.008**	**0.057**

Significant values in bold (p<0.05).

## Discussion

This study presents the first extensive analyses of the molecular ecology of *A. pelagicus* in the Pacific Ocean using both mitochondrial and nuclear molecular markers. Though an earlier study using mtDNA control region data [14], showed evidence for differences between eastern and western Pacific populations, the study was hampered by low sample size and geographic coverage, particularly a lack of samples from the central Pacific. The COI data from this study is largely concordant with this previous study, which is not surprising as the entire mtDNA molecule is a single locus. Both datasets found well-defined phylogenetic clades that were strongly separated by geography with samples on either side of the Pacific almost entirely composed of a single regionally specific clade. Samples collected in the Central Pacific represented a ∼50∶50 mix of these two clades offering a unique opportunity to test for reproductive isolation between these clades in an area of sympatry using nuclear markers.

A core task of this study was to evaluate and extend earlier findings by Eitner [13] and Trejo [14] regarding the pelagic thresher shark. By addition of multiple nuclear microsatellite loci and a broader geographic sampling we were able to test whether the distinct biogeographic pattern observed by Trejo [14] was maintained and evaluate the level of gene flow between eastern and western Pacific populations. We were also able to test whether the data better supported geographically separated populations or whether these distinct mtDNA clades represent reproductively isolated units. Microsatellite loci were mostly out of HWE in populations (i.e. Hawaii, Baja) that contained a mixture of animals with clade A and clade B haplotypes. When individuals within these populations were separated by mtDNA clade, deviations from HWE were mostly eliminated (Table 5). Structure analyses using the location prior to compare likelihoods of geographic versus mtDNA partitions indicated that grouping samples by mtDNA clade had a higher likelihood than grouping samples by geographic location (Table S2). However, in both cases, the K value was the same, with two as the most likely number of groups. Similarly, when AMOVA was run to compare these two hypothesized groupings, grouping samples by mtDNA clade explained three times the genetic variance as grouping samples geographically, while also reducing the within group variance. Structure results using geographic location as a prior resulted in two distinct groups, largely concordant with patterns observed with mtDNA haplotypes (Figure 4a). When the Structure analyses considered the mtDNA clade as the location prior there was very strong assignment of individuals to two distinct groups with little evidence for introgression (Figure 4b). Together these analyses support that the mtDNA clades first observed by Trejo are reproductively isolated from each other, even in areas of sympatry.

These results support the existence of two groups on separate evolutionarily trajectories, conforming to the concept of Evolutionarily Significant Units (ESUs). ESUs have been described based on the presence of reciprocal monophyly for organelle haplotypes (e.g. mitochondrial DNA) and significant divergence of allele frequencies at nuclear loci (e.g. microsatellites, [30]). In this study, we found two reciprocally monophyletic mtDNA clades with significant divergence of allele frequencies at microsatellite loci and a significant level of reproductive isolation between both clades in the Pacific Ocean based on the Structure analysis (Figure 4), both when this analysis was run using the geographic sampling location as prior or the COI clade as prior. The reason for this marked population division remains unclear.

Observed levels of H_O_ and H_E_ were similar across all locations and were generally higher than values observed in other oceanic species [6], [31], [32], but similar to those found in *I. oxyrinchus* [33]. The lower haplotype diversity in Taiwan could be a consequence of the sampling methodology as most samples came from a two sampling events, which could increase the chance of sampling related individuals. Though we sampled most of the geographic range of *A. pelagicus* in the Pacific, they are commonly found throughout the Indian Ocean so samples from the rest of the species range should be analyzed in order to assess the true population structure and genetic diversity within this ESU.

The overall findings in this study are in support of the assertion that a cryptic taxon exists within *Alopias* [13]. During the course of this study, several of the samples that Eitner [13] used in his study were evaluated for inclusion. Our initial analyses indicated that the *A. pelagicus* samples used in his study were most likely *A. superciliosus* and his unrecognized taxon was most likely *A. pelagicus*. Because of this discrepancy these samples were not included in this study.

Both our study and that of Trejo [14] identified regionally specific mtDNA clades on both sides of the Pacific. Samples from the central Pacific identified animals with haplotypes from both ESUs at high frequencies with little evidence for introgression in the microsatellite loci analyses. These results leave some new questions open; are both ESUs found year-round in this region or do they seasonally migrate here? Is there spatial or temporal separation between these groups that allow for reproductive isolation? Future movement studies should be conducted using electronic tags, especially in this zone of overlap.

## Conclusions

The results of this study provide important information to scientists, resource managers and governmental agencies regarding management and conservation of pelagic thresher sharks. The existence of two ESUs of *A. pelagicus* in the Pacific Ocean and the genetic differentiation presented here is the highest found in the literature for a large epipelagic shark. Moreover, considering the slow mutation rate of sharks compared to other vertebrates [34], the strong genetic differentiation found in *A. pelagicus* in the Pacific Ocean, and the almost nonexistent geneflow, this is likely an indicator of the existence of a cryptic species complex. Due to conflicts with the species concept found in the literature, we choose to leave our findings at the ESU level, although future research should include analyses of both morphological data and additional genetic markers. Regardless of the taxonomic label, the two ESUs described in this study warrant attention as they effectively form regional eastern and western Pacific populations which given their restricted geographic distribution makes them especially susceptible to overexploitation. These findings should be considered in management plans and initiatives such as the different National Actions Plans for the Conservation of Elasmobranch Species.

## Supporting Information

Figure S1 Plot of the second order rate of change of the likelihood (Δ*K*) showing the true value of K after testing *K* = 1–10 with 20 repetitions each.
(TIF)

Table S1 Name, PCR profile number, annealing temperature, reference or Genbank accession numbers for primers used in this study.
(DOC)

Table S2 Structure Likelihood values for multiple Ks with No Location Prior, Geographic Location Prior and mtDNA Clade Prior.
(DOC)
