# Supplementary figures and images for: Genetic Diversity and Population Structure of the Pelagic Thresher Shark (Alopias pelagicus) in the Pacific Ocean: Evidence for Two Evolutionarily Significant Units
Source: PLoS One. 2014 Oct 22;9(10):e110193. doi: 10.1371/journal.pone.0110193 (PMC4206417; doi:10.1371/journal.pone.0110193)

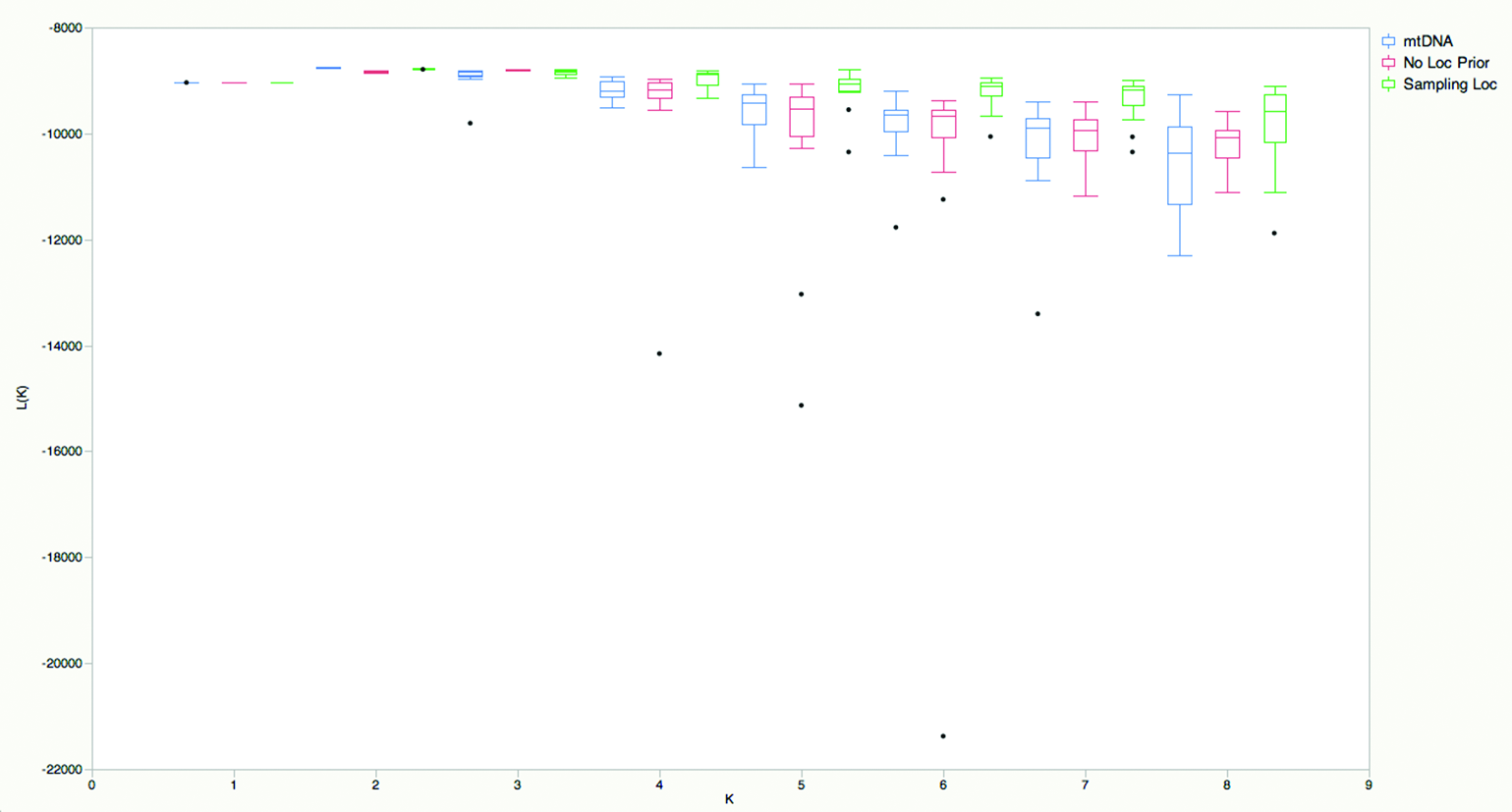

Supplement: Figure S1 — Plot of the second order rate of change of the likelihood (ΔK) showing the true value of K after testing K = 1–10 with 20 repetitions each. (TIF) [file pone.0110193.s001.tif]
