# Supplementary material for: Genetic Diversity and Population Structure of the Pelagic Thresher Shark (Alopias pelagicus) in the Pacific Ocean: Evidence for Two Evolutionarily Significant Units
Source: PLoS One. 2014 Oct 22;9(10):e110193. doi: 10.1371/journal.pone.0110193 (PMC4206417; doi:10.1371/journal.pone.0110193)
Supplement: Table S1 — Name, PCR profile number, annealing temperature, reference or Genbank accession numbers for primers used in this study. (DOC) [file pone.0110193.s002.doc]

| **Loci** | **PCR Profile** | **Annealing Temp. (°C)** | **Primer reference or accession number** |
| --- | --- | --- | --- |
| Iox-01 | 2 | 56 | Schrey and Heist (2002) |
| Iox-12 | 2 | 64 | Schrey and Heist (2002) |
| Iox-30 | 2 | 56 | Schrey and Heist (2002) |
| AV-H8 | 2 | 58 | KJ454444 |
| AV-H110 | 1 | 65 | KJ454442 |
| AV-H138 | 1 | 58 | KJ454443 |
| AV-I11 | 1 | 58 | KJ454447 |
| Iox-B3 | 2 | 62 | Schrey and Heist (2002) |
| Iox-M36 | 2 | 66 | KJ454435 |
| Iox-M115 | 2 | 68 | KJ454437 |
