# Supplementary material for: Genetic Diversity and Population Structure of the Pelagic Thresher Shark (Alopias pelagicus) in the Pacific Ocean: Evidence for Two Evolutionarily Significant Units
Source: PLoS One. 2014 Oct 22;9(10):e110193. doi: 10.1371/journal.pone.0110193 (PMC4206417; doi:10.1371/journal.pone.0110193)
Supplement: Table S2 — Structure Likelihood values for multiple Ks with No Location Prior, Geographic Location Prior and mtDNA Clade Prior. (DOC) [file pone.0110193.s003.doc]

|  | **Average Likelihood** | | |
| --- | --- | --- | --- |
| **K** | **No Loc Prior** | **Geographic Loc Prior** | **mtDNA Clade Prior** |
| 1 | -9038.34 ± 0.66 | -9038.20 ± 0.54 | -9038.15 ± 0.81 |
| 2 | -8829.32 ± 14.51 | -8774.73 ± 7.26 | -8756.49 ± 7.88 |
| 3 | -8793.28 ± 8.35 | -8848.87 ± 38.08 | -8936.23 ± 210.46 |
| 4 | -9426.70 ± 1125.09 | -8956.48 ± 160.74 | -9181.47 ± 184.97 |
| 5 | -10029.56 ± 1465.93 | -9137.45 ± 329.90 | -9538.94 ± 381.41 |
| 6 | -10407.14 ± 2623.26 | -9204.57 ± 290.78 | -9799.22 ± 545.62 |
| 7 | -10069.95 ± 460.52 | -9321.95 ± 358.24 | -10163.77 ± 867.99 |
| 8 | -10220.36 ± 413.64 | -9834.09 ± 735.29 | -10540.61 ± 863.89 |
